# Spatio-temporal variations in wheat aphid populations and their natural enemies in four agro-ecological zones of Pakistan

**DOI:** 10.1371/journal.pone.0222635

**Published:** 2019-09-30

**Authors:** Muhammad Faheem, Shafqat Saeed, Asif Sajjad, Su Wang, Abid Ali

**Affiliations:** 1 Department of Entomology, Faculty of Agricultural Sciences and Technology, Bahauddin Zakariya University, Multan, Pakistan; 2 CABI South East Asia, MARDI, Serdang, Selangor, Malaysia; 3 Department of Entomology, Faculty of Agriculture & Environmental Sciences, Muhammad Nawaz Shareef University of Agriculture, Multan, Pakistan; 4 Department of Entomology, University College of Agriculture and Environmental Sciences, The Islamia University of Bahawalpur, Bahawalpur, Pakistan; 5 Institute of Plant and Environment Protection, Beijing Academy of Agricultural and Forestry Sciences, Beijing, PR China; 6 Department of Entomology, Faculty of Agriculture, University of Agriculture, Faisalabad, Pakistan; Chinese Academy of Agricultural Sciences Institute of Plant Protection, CHINA

## Abstract

Aphids are major pests of wheat crop in Pakistan inflicting considerable economic losses. A better knowledge of landscape scale spatial distribution of aphids and their natural enemies could be used to improve integrated pest management programs. Therefore, the present study aimed to document spatio-temporal variations in populations of wheat aphids and their natural enemies in Pakistan. The 2-year survey study was carried out at ten experimental farms located in five districts of four contrasted agro-ecological zones of eastern Pakistan (Punjab area) i.e. District Chakwal in arid zone, Gujranwala in rice-cropped zone, Faisalabad in central mixed-cropped zone, and Khanewal and Multan in cotton-cropped zone. The dominant aphid species i.e. *Schizaphis graminum*, *Rhopalosiphum padi*, *R*. *maidis* and *Sitobion avenae* varied significantly among the five districts surveyed. The population of *S*. *graminum* was observed more abundant in arid, *R*. *padi* in rice, *S*. *avenae* in aird and rice, and *R*. *maidis* in cotton-I zones. Aphids ended their population dynamics on 25^th^ March in central mixed-cropped zone and 12^th^ April in other three zones. Various species of natural enemies, mainly *Coccinella septumpunctata*, *C*. *undecimpunctata*, *Menochilus sexmaculata*, *Chrysoperla carnea*, Syrphidae and parasitoid mummies were inconsistently observed in four agro-ecological zones. The population of *C*. *septumpunctata*, was observed more abundant in rice zone, *C*. *undecimpunctata* and *C*. *carnea* in cotton-I and arid zones, *M*. *sexmaculata* in cotton-I and II zones, Syrphidae in cotton-I zone and parasitoid mummies in rice and arid zones. There were no clear relationships between aphid and the natural enemy populations. The present study may serve as a baseline regarding distribution of wheat aphids and their natural enemies and the results provided insights for further studies on the potential top-down (natural enemies) versus bottom-up (fertilization and irrigation regimes) forces in management of wheat aphids in eastern Pakistan.

## Introduction

Wheat (*Triticum aestivum* L.) is Pakistan’s staple diet and accounts for 10 percent of value added in agriculture and contributes over two percent to the country’s GDP [1]. It has been cultivated on over nine million ha with production of 26.3 million tons of grains with an average yield of 2,893 kg per ha in 2017–18 [2]. The average wheat yield is even lower than the neighbouring countries like India and Bangladesh i.e. 2,962 kg per ha and 4357 kg per ha, respectively [1]. Several factors are responsible for the low yield of wheat in Pakistan like varieties [3], sowing time [4], improper inputs as water and fertilizers [5], weeds [6] rainfall and insect pests. Among insect pest, wheat aphids are gradually attaining regular pest status in Pakistan [7]. Aphids reduce yield by direct damages i.e. sap sucking from plant parts, yellowing and reduced grain size [8, 9], and indirect damages through transmitting plant virus [10, 11]. The direct wheat yield losses may reach up to 35–40% and indirect losses up to 20–80% due to aphids [12].

Several species of aphids have been reported from various agro-ecological zones of Pakistan. *Rhopalosiphum padi*, *Sitobion avenae* and *Schizaphis graminum* have been reported from Khayber Pakhtun Khaw (KPK) province while, *Macrosiphum miscanthi*, *Sipha mydis* and *Sipha elegans* from northern hilly areas (temperate climate) [13–15]. In addition to *S*. *graminum* and *M*. *miscanthi*, *Aphis maidis* has been recorded from the plains of the Punjab [16, 17]. Punjab province has been divided into eleven agro-ecological zones on the basis of climates supporting particular cropping patterns [18]. The population dynamic pattern of aphids varies in different agro-ecological zones of Pakistan due to variation in climatic conditions. They appeared and departed earlier in the warmer areas and later in the colder areas [15]. Population fluctuations have also been recorded among consecutive years within a location [15, 19–21]. Multiple factors affect spatial distribution of aphids including climatic conditions and some biotic factors such as quality of host plants, dispersal efficacy of aphids and natural enemies [22, 23].

The infestation of cereal aphids can be reduced by applying various biological, cultural and chemical control strategies [24]. Pesticides are being used against aphids on wheat, known to have negative effects on key beneficial arthropods [25, 26], including natural enemies of aphids e.g. affect the oviposition behaviour and functional response of parasitosis i.e. *Aphidius ervi*, *Diaeretiella rapae*, *A*. *matricariae*, *Aphytis melinus*, *Lysiphlebus fabarum* [27–31], besides promoting selection of resistances in pest populations [32, 33]. Knowledge of spatial distribution of aphids at the field scale can be used to create or improve pest monitoring procedures and adjust pesticide application programs, as well as to effectively plan augmentative releases of biological control agents [34–36]. Temporal distribution of sucking insect pests and their natural enemies in different crops have been studied worldwide [37–42]. However, in Pakistan, there is few information on spatial and temporal variations in natural enemies of wheat aphids, notably with special reference to their potential top-down effect, their present status and distribution patterns.

Therefore, there is need to conduct a study to investigate tempo-spatial variations in populations and seasonal dynamics of various aphid species and their natural enemies across the four agro-ecological zones in eastern Pakistan. These findings could serve as a baseline for using natural enemies in optimized integrated aphid management strategies in plain areas of the Pakistan.

## Materials and methods

### Study area

**Fig 1:**
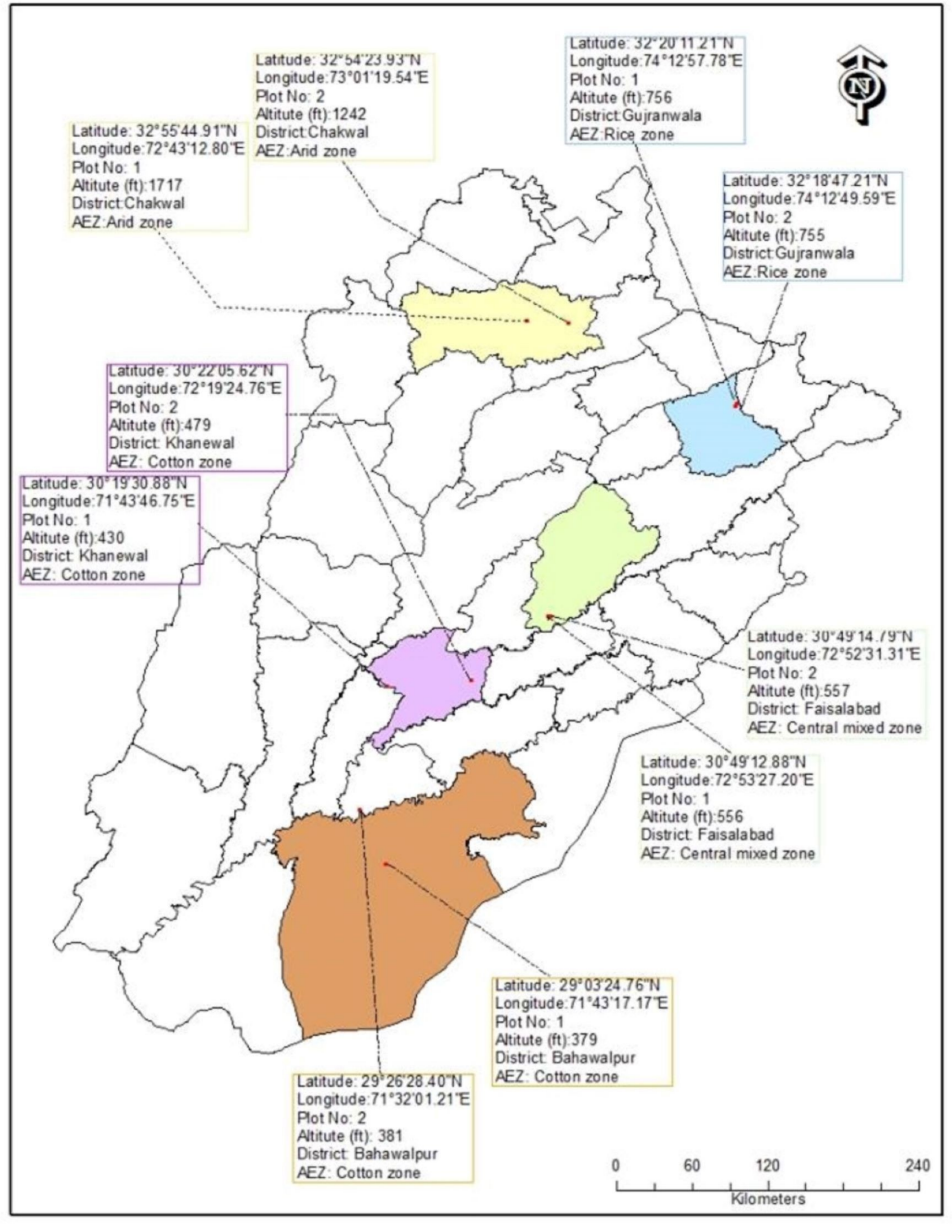
The map of experimental plots in five districts of Punjab province of Pakistan.

The study was conducted at ten experimental farms without use of pesticides in four agro-ecological zones of Punjab, Pakistan during two consecutive wheat growing seasons in 2010 and 2011 (Fig 1). The agro-ecological zones, i.e., cotton zone, central mixed zone, rice zone and arid zone [18] are established on the basis of average annual rainfall i.e. 156, 446, 800 and 900 mm, respectively [43]. District Khanewal and Bahawalpur were selected from cotton zone (we regarded Khanewal as cotton zone I and Bahawalpur as cotton zone II), Faisalabad from central mixed zone, Gujranwala from rice zone and Chakwal from arid zone. In each district, two experimental plots (2 ha for each) were selected with minimum 10 km buffer from each other having wheat crops and other vegetation (Table 1, Fig 1). To overcome the marginal effects, each study plot was surrounded by wheat fields by at least 1 ha. Commonly grown wheat variety ‘Sahar’ was planted in two 2-ha plots at each agro-ecological zone through drill sowing method at the sowing rate of 125 kg/ha from October 15^th^ to 31^st^ in both growing seasons (2010–2011). All agronomic practices were done according to local recommendations.

**Table 1:** Detail for the distribution and selection of experimental plots in four different ecological zones during 2010 and 2011 in Punjab province of Pakistan.

Agro-ecological Zone	Rainfall (mm)	Districts	Plot No	Latitude	Longitude	Altitude (ft)
Arid zone	900	Chakwal	1	32°55'44.91"N	72°43'12.80"E	1717
			2	32°54'23.93"N	73°01'19.54"E	1242
Rice zone	800	Gujranwala	1	32°20'11.21"N	74°12'57.78"E	756
			2	32°18'47.21"N	74°12'49.59"E	755
Central mixed zone	446	Faisalabad	1	30°49'12.88"N	72°53'27.20"E	556
			2	30°49'14.79"N	72°52'31.31"E	557
Cotton zone I	156	Khanewal	1	30°19'30.88"N	71°43'46.75"E	430
			2	30°22'05.62"N	72°19'24.76"E	479
Cotton zone II		Bahawalpur	1	29°03'24.76"N	71°43'17.17"E	379
			2	29°26'28.40"N	71°32'01.21"E	381

### Sampling

Weekly visual observations of aphids and their natural enemies were started from 8^th^ February to 12^th^ April during both the seasons. Three hundred and forty eight sampling points were selected in each of 2-ha plot in zigzag fashion on each observation day and completed within a week from all ten locations. On each sampling point, three consecutive tillers of a wheat plant were randomly selected and visually observed for counting species of aphids (*Schizaphis graminum*, *Rhopalosiphum padi*, *Rhopalosiphum maidis* and *Sitobion avenae*) and their natural enemies (*Coccinella septumpunctata*, *C*. *undecimpunctata*, *Menochilus sexmaculata*, *Chrysoperla carnea*, syrphidae and parasitoid mummies). Aphid species were morphologically identified using the identification keys developed by the University of Idaho [44]. Only the visually identifiable mummies e.g. having change in colour and swollen were counted, while already hatched mummies were excluded from the counts. In the case of heavy infestation of aphids, tillers were clipped and put in paper bags, which were then preserved in ice chest boxes for later counting in the laboratory [45].

### Statistical analyses

Prior to analysis, data were checked for their conformance to the normal distribution using Kolmogrov-Simirnov normality test. Per tiller populations of aphids and natural enemies among the five locations were compared per species (or group of species) using Kruskal-Wallis tests. The means of ranks were compared using Dunn’s post hoc test. To compare the difference in the means of population of aphids and natural enemies between the two years, T-test (two tailed, paired sample) was applied. The relationship between natural enemy and aphid density was tested using linear regression analysis. The data of all aphids and natural enemies species were pooled to compare the relative proportion of natural enemies and aphids in four agro-ecological zones during 2010 and 2011. XLSTAT computer software was used for all analysis [46].

## Results

### Population dynamics of aphids

**Fig 2:**
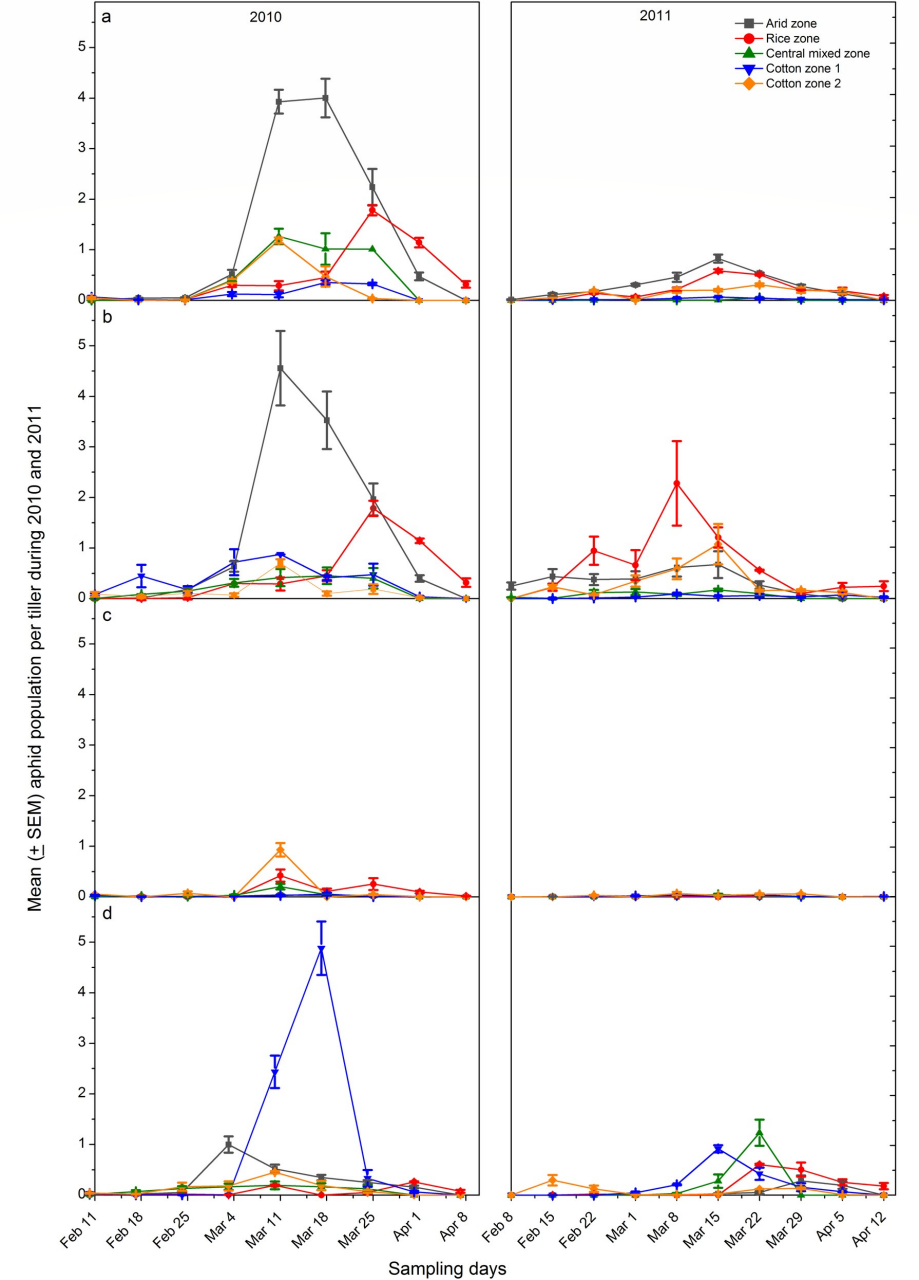
Seasonal fluctuation of different aphid species in four ecological zones of Punjab, Pakistan during 2010 and 2011. (a) *Schizaphis graminum*, (b) *Rhopalosiphum padi*, (c) *R*. *maidis* and (d) *Sitobion avenae*. Error bars show ± standard error of means.

In 2010 season, the population of all four aphid species did not differ significantly among the five districts (*S*. *graminum*: KW = 3.27, *P* = 0.51, *d*.*f*. = 4; *R*. *padi*: KW = 2.97, *P* = 0.56, *d*.*f*. = 4; *R*. *maidis*: KW = 3.92, *P =* 0.42, *d*.*f*. = 4; *S*. *avenae*: KW = 1.55, *P* = 0.82, *d*.*f*. = 4) (Table 2). All the aphid species were present from 18^th^ February to 4^th^ March. The peak activity period of aphids lasted from 11^th^ to 18^th^ March. During this period, the aphid species, *S*. *graminum* and *R*. *padi* were the most abundant in arid zone while *R*. *maidis* and *S*. *avenae* in cotton zone II and cotton zone I, respectively (Fig 2).

**Table 2:** Means of ranks of population of aphids and their natural enemies as computed by Dunn’s post hoc test at alpha 0.05.

Seasons	Species	Cotton zone II	Arid zone	Mixed zone	Rice zone	Cotton zone I
**2010**	*S*. *graminum*	19.66 a	28.66 a	22.44 a	25.00 a	19.22 a
	*R*. *padi*	18.22 a	26.66 a	19.88 a	24.16 a	26.05 a
	*R*.*maidis*	22.61 a	19.44 a	24.00 a	29.72 a	19.22 a
	*S*. *avenae*	23.27 a	27.38 a	22.27 a	20.05 a	22.00 a
**2011**	*S*. *graminum*	28.65 ab	35.10 a	11.55 b	32.35 a	19.85 ab
	*R*. *padi*	27.75 ab	31.95 ab	16.65 b	36.25 a	14.90 b
	*R*. *maidis*	30.45 ab	13.25 b	25.35 ab	26.60 ab	31.85 a
	*S*. *avenae*	27.90 a	21.65 a	19.05 a	29.10 a	29.80 a
**2010**	*C*. *septumpunctata*	16.5 a	17.0 a	20.83 a	31.88 a	28.77 a
	*C*. *undecimpunctata*	22.66 a	20.55 a	23.22 a	20.55 a	28.0 a
	*M*. *sexmaculata*	18.50 a	23.61 a	25.77 a	18.50 a	28.61 a
	*C*. *carnea*	19.77 a	27.83 a	20.44 a	17.50 a	29.44 a
	Syrphidae	15.22 bc	12.88 c	21.22 abc	29.55 ab	36.11 a
	Mummies	13.50 b	18.94 ab	26.33 ab	30.16 a	26.05 ab
**2011**	*C*. *septumpunctata*	22.7 ab	28.7 ab	14.00 b	35.20 a	26.90 ab
	*C*. *undecimpunctata*	23.15 a	31.55 a	23.35 a	28.45 a	21.00 a
	*M*. *sexmaculata*	22.70 a	21.20 a	20.40 a	18.50 a	20.40 a
	*C*. *carnea*	22.25 a	29.65 a	22.45 a	24.25 a	28.90 a
	Syrphidae	20.30 a	27.20 a	18.70 a	30.30 a	31.00 a
	Mummies	20.50 a	30.90 a	25.00 a	28.45 a	22.65 a

Means of ranks sharing similar lettering within a row are non-significant at 0.05% level.

In 2011 season, except for *S*. *avenae*, the population of *S*. *graminum*, *R*. *padi* and *R*. *maidis* varied significantly (KW = 17.81, *P*<0.001, *d*.*f*. = 4; KW = 16.68, *P*<0.001, *d*.*f*. = 4; KW = 11.12, *P* = 0.03, *d*.*f*. = 4; respectively) among the five districts (Table 2). All aphid species started their activity from 15^th^ February. Peak abundance of all aphid species was recorded from 8^th^ to 22^nd^ March followed by a sharp or gradual decline until 12^th^ April (Fig 2). The abundance of the species varied greatly among different zones during peak activity period (8^th^ to 22^nd^ March). *Schizaphis graminum* and *R*. *padi* were the most abundant at arid and rice zones, respectively. *Rhopalosiphum maidis* was the most abundant at cotton zone II whereas it was the least abundant on 22^nd^ March during 2011. *Sitobion avenae* was the most abundant at cotton zone I on the 22^nd^ March (Fig 2).

### Population dynamics of natural enemies

**Fig 3:**
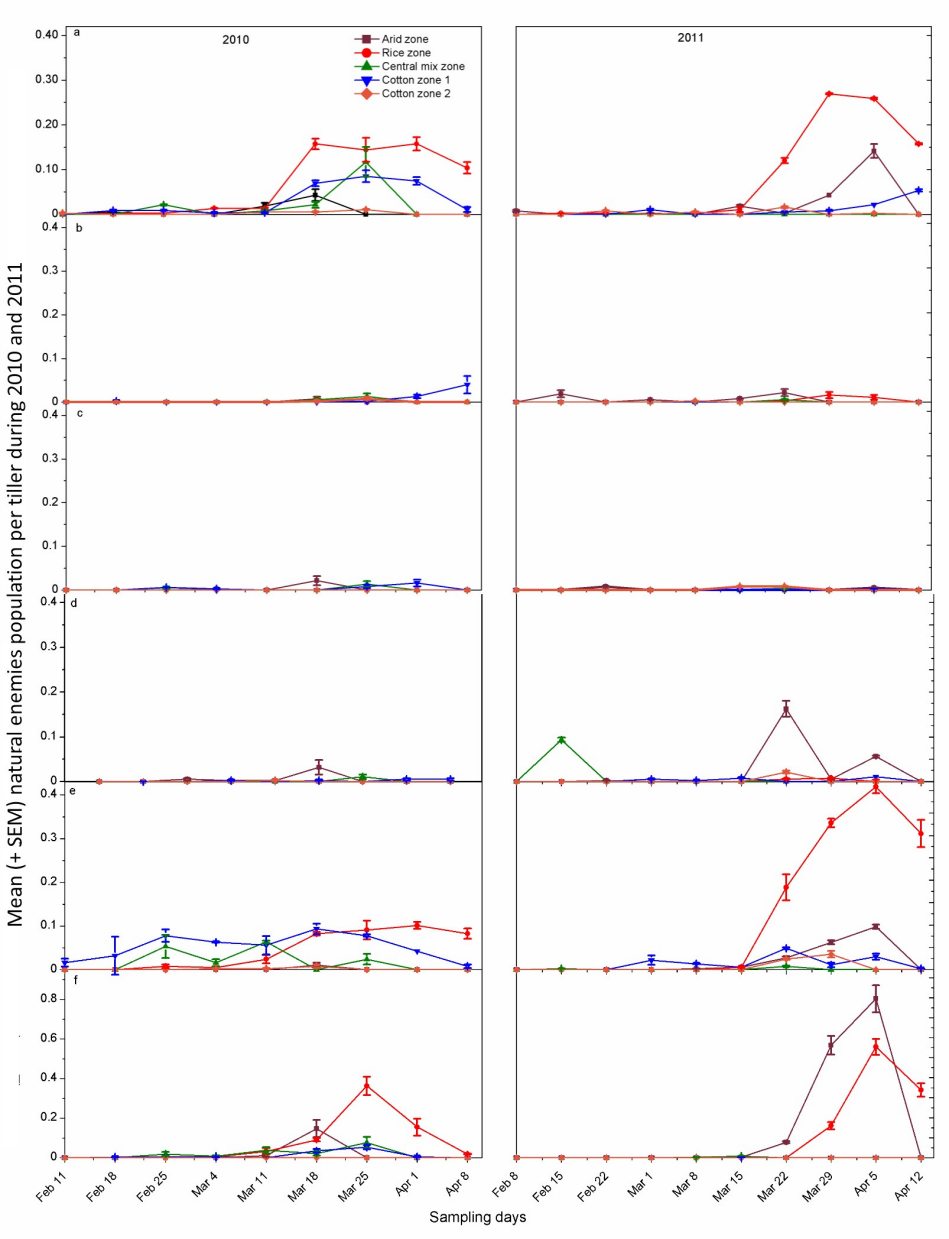
Seasonal fluctuation of different natural enemies in four ecological zones of Punjab, Pakistan during 2010 and 2011. (a) *Coccinella septumpunctata*, (b) *C*. *undecimpunctata*, (c) *Menochilus sexmaculata*. (d) *Chrysoperla carnea*, (e) Syrphidae and (f) Mummies. Error bars show ± standard error of means.

In 2010 season, the population of syrphids and mummies varied significantly (KW = 22.12, *P*<0.001, *d*.*f*. = 4; KW = 11.54, *P* = 0.02, *d*.*f*. = 4, respectively) among the five districts of four agro-ecological zones. The Dunn’s post hoc test showed the maximum mean of ranks for Syrphidae in cotton zone I and the minimum for arid zone. In the case of mummies, the maximum mean of ranks was recorded for rice zone and the minimum for cotton zone II (Table 2). Population dynamics of all the natural enemies started to increase from the 11^th^ March and attained peak from 18^th^ March to 1^st^ April (Fig 3A–3F).

In 2011 season, except for *C*. *septumpunctata* (KW = 13.79, *P* = 0.01, *d*.*f*. = 4), all the other five natural enemies did not differ significantly among the five districts (all *P*>0.05, *d*.*f*. = 4). The Dunn’s post hoc test showed the maximum mean of ranks of *C*. *septumpunctata* in rice zone and the minimum in the mixed zone (Table 2). The three types of natural enemies, *C*. *septumpunctata*, syrphid and mummies peaked from 29^th^ March to 5^th^ April. *Coccinella undecimpunctata* and *M*. *sexmaculata* abundance were relatively low during the season (Fig 3A–3F).

**Fig 4:**
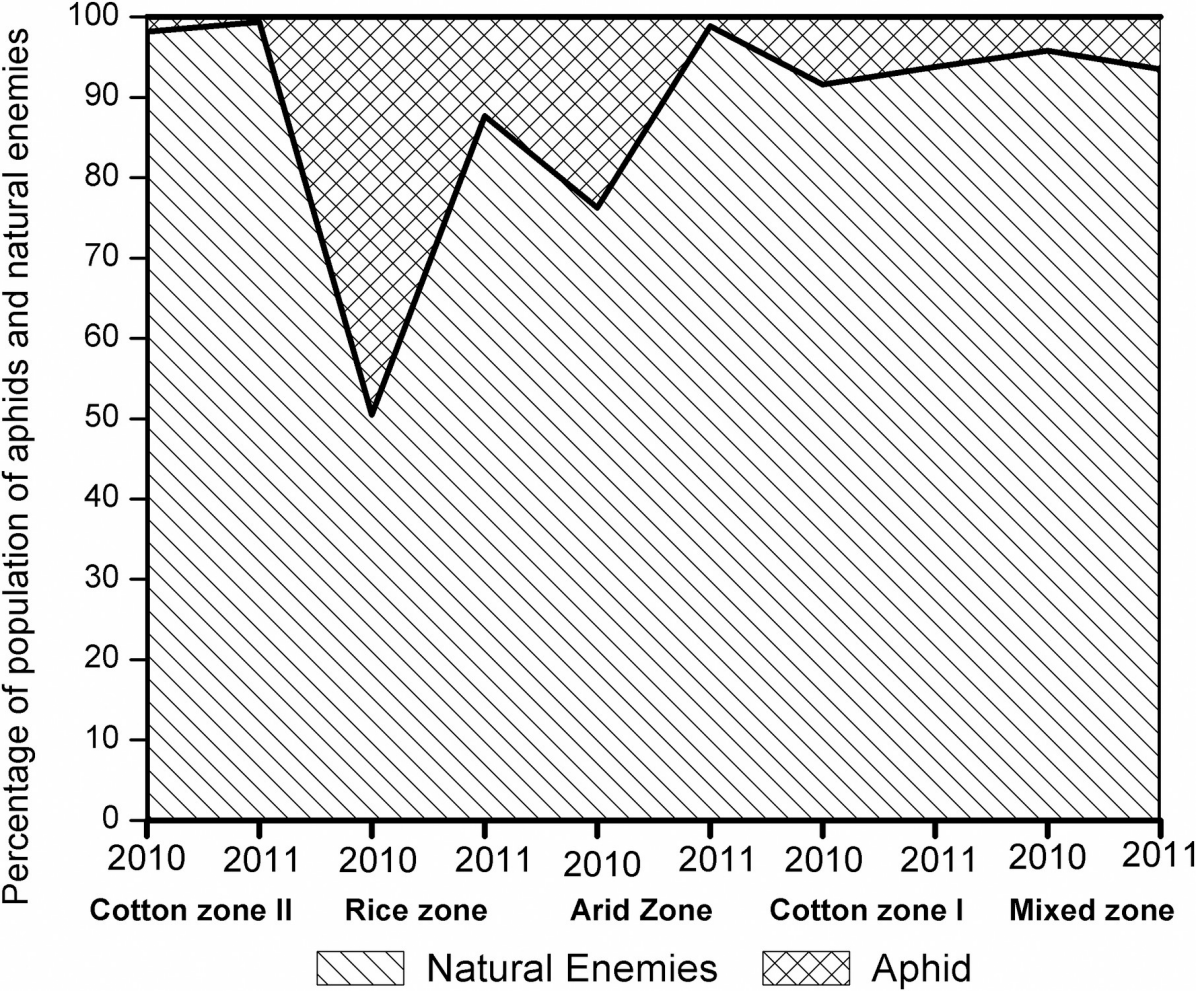
Relative proportion of cumulative aphids and natural enemies species in four ecological zones of Punjab, Pakistan during 2010 and 2011. Diagonal line area = Aphids, Diamond grid area = Natural enemies.

Overall seasonal comparison of aphids revealed that *S*. *graminum* and *R*. *maidis* were more abundant in 2010 than those in 2011(T = 2.81, *P* = 0.006, *d*.*f*. = 93 and T = 2.13, *P* = 0.036, *d*.*f*. = 93, respectively). There was no significant relationship between populations of the aphids and their natural enemies in both seasons (linear regressions: both *P*>0.05). The relative proportion between cumulative aphids and natural enemies population in five zones showed that the highest relative proportion of natural enemies to aphids was observed in rice and arid zones during 2010, and rice and mix zones during 2011. The natural enemies were relatively more abundant during 2010 than 2011 (Fig 4).

## Discussion

The populations of aphid species did not differ significantly among the five districts in season 2010 except for *S*. *avenae*, which differed significantly in season 2011. Spatial variation of aphids and other insect pest species is predicted by climatic conditions and some biotic factors such as dispersal efficiency, host plants genotype identity and natural enemies [22, 23, 47]. The migration is another factor which may affect aphid populations while temperature is a dominant factor affecting aphid migration phonologies [48]. Understanding spatial variability of aphids in wheat agro-ecosystems using certain models can potentially facilitate site specific application of pesticides, decreasing the amount and cost of pesticides, environmental pollution and pesticide exposure to farmers while increasing the biological control agents [49]. A recent preliminary study suggests that forecasting of aphid epidemics can be more reliable within the radius of only 10 km [50]. However, landscape scale studies can create or improve pest monitoring procedure and pesticide application programs at regional level [36].

*Sitobion avenae* was the most abundant in arid and rice zones whereas *R*. *padi* in rice zone. On the other hand, population densities of *R*. *maidis* were the most abundant in cotton zone I. The distribution patterns of wheat aphid species have not been reported so far in Punjab province of Pakistan. It is known that abiotic factors such as temperature, rainfall, fertilization and irrigation are crucial for regulating arthropod populations [51–58]. Both the arid and rice zones of Punjab are relatively colder with high altitude and rainfall while cotton zone is relatively warmer with low altitude and rainfall. The mean monthly temperatures of arid, rice and cotton zone are 20°C, 25°C and 30°C, respectively while annual rainfall is 40, 20 and 10 inches, respectively [59].

In present study, the peak activity period of all the aphid species lasted from 11^th^ to 18^th^ March in 2010 while from 8^th^ to 22^nd^ March in 2011 followed by a sharp or gradual decline until 12^th^ April. Khan (2005) [15] reported seasonal trends of wheat aphids from Khayber Pakhtun Khaw (KPK) province i.e. aphids appear and depart earlier in warmer districts and vice versa. However, we did not see any remarkable difference in aphids’ dynamics among the warmer and colder districts of Punjab in this study. This might be due to overall colder climate of KPK compared to the districts of Punjab. Mühlenberg and Stadler (2005) [60] reported similar findings that the warmer and drier conditions at the low altitude site resulted in earlier aphid multiplication in spring compared with high altitude site, where aphid population got their peak few weeks later.

Seasonal dynamics of aphids followed almost similar pattern in both growing seasons (2010 and 2011), i.e. the population started to grow from the 15^th^ February, peaked during last fortnight of March followed by a sharp or gradual decline until the 12^th^ April. Seasonal dynamic pattern of aphids varied across latitude as several previous studies have documented slightly different trends in Multan and Peshawar districts. Aslam et al. (2004) [16] reported that in Multan population appeared on the 3^rd^ week of January and increased exponentially and peaked on the 3^rd^ week of March followed by a sudden decline after the 4^th^ week of March until the 1^st^week of April. Similarly, Khattak et al (2007) [19] reported from Peshawar that population of aphids started to build up from the 2^nd^ week of January and attained its peak on the first two weeks of March. Another study from Peshawar suggested that aphid population started to increase in the last week of December, remained low populations in January and peaked in the 1^st^ week of March [20].

Population dynamics of natural enemies attained their peak from the 18^th^ March to 1^st^ April in 2010 while 29^th^ March to 5^th^ April in 2011. Similar findings have been reported by Saleem et al. (2009) [20] from Peshawar documenting the peak population density of coccinellids in 4^th^ week of March, syrphids in the 3^rd^ week of March, chrysopids in the 2^nd^ week of April and parasitoids in the 3^rd^ week of March. The fluctuation of natural enemies was more abrupt than aphids in 2010 season compared to 2011 season. The populations of Syrphidae and mummies varied significantly among the five districts in season 2010 while only *C*. *septumpunctata* varies significantly in season 2011. Syrphid flies are efficient predators of aphids and exponentially decrease their population [61, 62]. The relationship between predators and prey densities has been a central theme in ecological theories [63, 64]. In agricultural system, predator-prey interactions play a key role in suppressing herbivore prey (often regarded as pests), which is suggested to be examined aiming to reduce crop damage and increase crop yield [40, 65–69]. Ideally, predators should respond numerically by aggregating attacks with high prey densities for an effective biological control or switch among different prey species pending on their relative abundance [70–73]. However, such an ideal scenario has rarely been supported by empirical data. In present study we found no correlation between the population of aphids and natural enemies in both years. Several factors may explain such discrepancies including differences in spatial scale at which observations are made [74], behavioral differences among species, varying habitat features (i.e. shelters and nesting sites), availability & distribution of alternate prey [23, 37, 75], and genotypic diversity that can influence the effects of spatial processes on the plant-herbivore interactions [47]. These factors are also important with view point of aphids forecasting as they determine the reinvasion pattern in the following season [76].

The present study for the first time established a baseline data on distribution and seasonal population dynamics of wheat aphids and their natural enemies in four agro-ecological zones in eastern Pakistan. Further research is warranted to examine (i) the top-down vs. bottom-up forces in wheat agro-ecosystems in order to develop sustainable IPM, and (ii) side effects of commonly used insecticides on the behavior and physiology of those potential natural enemies.
